# SENESCENCE AND INFLAMMATION

**DOI:** 10.1093/geroni/igac059.780

**Published:** 2022-12-20

**Authors:** Marissa Schafer

## Abstract

Cell senescence and inflammation are interconnected mediators of aging and age-related disease. Recent advances in molecular and cellular profiling methods and research models are aiding in our ability to decipher mechanisms through which senescent cells drive inflammatory dysfunction, and inversely, to discover mechanisms through which aging immune cells may drive senescence, inflammation, and pathology. This symposium will feature exciting advances that span the emerging conceptual framework of how senescence and inflammation influence mammalian aging. Dr. Birgit Schilling will discuss the use of advanced mass spectrometric methods for profiling the senescent cell proteome, which reveal new insights into protein pathways and mechanisms of aging and disease. Dr. Matt Yousefzadeh will share how endogenous DNA damage can invoke cellular senescence, which enhances inflammation in both a cell autonomous and non-autonomous manner to drive tissue dysfunction and impact health. Dr. Daniel Tyrrell will discuss discovery of a novel population of age-associated CD8 T-cells that enhance local tissue senescence and inflammation and promote atherosclerosis. Dr. Xu Zhang will share a characterization of senescent cells in skeletal muscle using single-cell RNA-sequencing and the potential recruitment of immune cells by senescent fibroadipogenic progenitors. Dr. Marissa Schafer will discuss cell senescence as a mediator of age-related brain inflammatory cell composition and senescent cell targeting as a strategy to prevent cognitive decline. Importantly, discoveries discussed in this symposium may reveal new avenues for therapeutic development, to ultimately improve human healthspan.

